# Trends in Interregional Travel to Shopping Malls and Restaurants Before and After Differential COVID-19 Restrictions in the Greater Toronto Area

**DOI:** 10.1001/jamanetworkopen.2021.23139

**Published:** 2021-08-31

**Authors:** Jean-Paul R. Soucy, Amir Ghasemi, Shelby L. Sturrock, Isha Berry, Sarah A. Buchan, Derek R. MacFadden, Kevin A. Brown

**Affiliations:** 1 Division of Epidemiology, Dalla Lana School of Public Health, University of Toronto, Toronto, Ontario, Canada; 2 Communications Research Centre Canada, Ottawa, Canada; 3 Public Health Ontario, Toronto, Ontario, Canada; 4 Ottawa Hospital Research Institute, Ottawa, Ontario, Canada

## Abstract

This cohort study examines whether the implementation of differentially timed restrictions in a highly interconnected metropolitan area was associated with increased interregional travel.

## Introduction

In the fall of 2020, the government of Ontario, Canada, adopted a 5-tier, regional framework of public health measures for the COVID-19 pandemic in its 34 public health regions.^1^ The goal of nonpharmaceutical interventions was to suppress transmission by reducing contact rates, which can be indirectly assessed using mobility data. Five of the 6 most populous health regions in Ontario are located in the Greater Toronto Area: Toronto (3.0 million), Peel (1.5 million), York (1.2 million), Durham (0.7 million), and Halton (0.6 million). The urban core of Toronto and Peel is a perpetual hotspot for COVID-19^2^ and remains highly interconnected with the peripheral regions of York, Durham, and Halton.

Toronto and Peel were the first regions in Ontario to enter the highest restriction tier (ie, lockdown) during the second wave of COVID-19. On November 23, 2020, Toronto and Peel closed restaurants to in-person dining and limited nonessential businesses, including shopping malls, to curbside pickup. York entered lockdown on December 14, 2020, followed by the rest of the province, including Durham and Halton, on December 26, 2020. In this cohort study, we examine whether the implementation of differentially timed restrictions in a highly interconnected metropolitan area was associated with increased interregional travel, potentially driving further transmission of SARS-CoV-2.

## Methods

This cohort study received ethical approval from the University of Toronto research ethics board through the Ontario COVID-19 Modeling Consensus Table. Informed consent was waived because data were anonymous, and the study posed minimal risk. This study follows the Strengthening the Reporting of Observational Studies in Epidemiology (STROBE) reporting guideline.

We used anonymized mobile device data from Veraset representing 154 089 unique devices (3.4% of the population) to analyze patterns of travel by residents of regions in the urban core (Toronto and Peel) to shopping malls and restaurants in peripheral regions in the week before the November 23 lockdown compared with the week after the lockdown (eFigure in the Supplement). Restaurants and shopping malls are both important settings for transmission risk.^3,4^ A device’s home region for a given month was identified as where it spent most of its time during that month. The proportion of devices in the data set that visited malls or restaurants was multiplied by the population of the region (2019 estimates)^5^ to estimate the actual number of visitors. We also measured visits by residents of Toronto and Peel to shopping malls in York relative to a baseline calculated for each day of the week from January 1 to February 5, 2020. Neighborhood sociodemographic characteristics of devices captured in the Veraset sample are contrasted with the general population of Toronto and Peel in the eTable in the Supplement.

One-sided *P *values were calculated using the bayesian posterior distribution of a structural time series fit to the preintervention daily data. Statistical significance was set at *P* < .05, and data analysis was performed between January 2021 to June 2021 using the statistical package R version 4.0.2 (R Foundation for Statistical Computing).

## Results

Residents of Toronto and Peel took fewer trips to shopping malls and restaurants in the week following lockdown (shopping malls: Toronto, −15.3% [95% CI, −28.5 to −5.4]; Peel, −18.2% [95% CI, −30.0 to −4.7]; restaurants: Toronto, −16.9% [95% CI, −28.8 to 0.0]; Peel, −20.2% [95% CI, −32.1 to −6.7]) (Table). During the same time, there was a significant increase in trips to shopping malls in peripheral regions by residents of the regions in lockdown (Toronto: +40.7% [95% CI, 27.0 to 56.6]; Peel: +65.5% [95% CI, 54.2 to 81.7]); however, visits to peripheral regions were still well below historical means (Figure). Visits to restaurants in peripheral regions did not decrease (Toronto: +6.3% [95% CI, −8.0 to 23.6]; Peel: +11.8% [95% CI, −6.0 to 20.9]).

**Table.** Estimated Number and Percent Change of Toronto and Peel Residents Visiting Shopping Malls and Restaurants in Other Regions of Ontario, One Week Before and After Lockdown (November 23, 2020)^a^

Region of travel	Shopping malls, No. (95% CI)		Change, % (95% CI)	Restaurants, No. (95% CI)		Change, % (95% CI)
	Before	After		Before	After	
**Residents of Toronto**						
Urban core						
Toronto	80 377 (71 220 to 90 715)	56 198 (48 035 to 65 788)	−30.1 (−41.6 to −19.8)	59 996 (52 167 to 69 010)	47 139 (39 762 to 55 944)	−21.4 (−33.7 to −5.1)
Peel	6273 (4098 to 9639)	5131 (3054 to 8641)	−18.2 (−38.6 to −2.9)	2437 (1235 to 4831)	1807 (790 to 4257)	−25.9 (−39.2 to −14.7)
Total	86 650 (75 318 to 100 354)	61 329 (51 089 to 74 429)	−29.2 (−43.0 to −19.1)	62 433 (53 402 to 73 841)	48 946 (40 552 to 60 201)	−21.6 (−34.0 to −7.1)
Peripheral regions						
York	12 814 (9473 to 17 345)	19 283 (14 771 to 25 225)	50.5 (38.7 to 61.2)	7742 (5272 to 11 401)	8544 (5699 to 12 821)	10.4 (−4.7 to 32.8)
Durham	3776 (2195 to 6548)	4072 (2303 to 7275)	7.8 (−11.0 to 28.6)	1765 (814 to 3907)	1351 (531 to 3598)	−23.5 (−44.5 to −1.4)
Halton	1736 (841 to 3821)	2424 (1184 to 5097)	39.6 (−7.4 to 92.6)	499 (108 to 2059)	744 (201 to 2709)	49.1 (20.4 to 82.4)
Total	18 326 (12 509 to 27 714)	25 779 (18 258 to 37 597)	40.7 (27.0 to 56.6)	10 006 (6194 to 17 367)	10 639 (6431 to 19 128)	6.3 (−8.0 to 23.6)
Other regions	1670 (250 to 7941)	3179 (1025 to 10 597)	90.4 (64.7 to 119.1)	2385 (531 to 9795)	2613 (665 to 10 590)	9.6 (−16.2 to 33.4)
Overall	106 646 (88 077 to 136 009)	90 287 (70 372 to 122 623)	−15.3 (−28.5 to −5.4)	74 824 (60 127 to 101 003)	62 198 (47 648 to 89 919)	−16.9 (−28.8 to 0.0)
**Residents of Peel**						
Urban core						
Toronto	9943 (7251 to 13 670)	5666 (3618 to 8912)	−43.0 (−64.1 to −26.2)	6549 (4456 to 9677)	5296 (3348 to 8442)	−19.1 (−39.6 to 1.2)
Peel	53 324 (46 434 to 61 240)	37 255 (31 230 to 44 472)	−30.1 (−43.3 to −14.6)	22 152 (17 877 to 27 460)	16 101 (12 319 to 21 078)	−27.3 (−39.4 to −10.8)
Total	63 267 (53 685 to 74 910)	42 921 (34 848 to 53 384)	−32.2 (−42.5 to −20.2)	28 701 (22 333 to 37 137)	21 397 (15 667 to 29 520)	−25.4 (−38.9 to −7.2)
Peripheral regions						
York	3816 (2297 to 6372)	6508 (4268 to 9945)	70.5 (55.5 to 89.1)	1572 (732 to 3449)	1599 (728 to 3663)	1.7 (−15.9 to 24.5)
Durham	289 (96 to 909)	40 (2 to 232)	−86.2 (−151.8 to −24.5)	227 (42 to 997)	42 (2 to 239)	−81.5 (−155.3 to −29.6)
Halton	5260 (3402 to 8153)	8948 (6274 to 12 820)	70.1 (53.5 to 85.1)	3373 (1972 to 5810)	4140 (2461 to 7017)	22.7 (0.0 to 38.3)
Total	9365 (5795 to 15 434)	15 496 (10 544 to 22 997)	65.5 (54.2 to 81.7)	5172 (2746 to 10 256)	5781 (3191 to 10 919)	11.8 (−6.0 to 20.9)
Other regions	3257 (956 to 11 516)	3690 (1105 to 12 823)	13.3 (−2.4 to 32.8)	2029 (468 to 8277)	1461 (343 to 5854)	−28.0 (−49.8 to −3.6)
Overall	75 889 (60 436 to 101 860)	62 107 (46 497 to 89 204)	−18.2 (−30.0 to −4.7)	35 902 (25 547 to 55 670)	28 639 (19 201 to 46 293)	−20.2 (−32.1 to −6.7)

^a^ 95% CIs were calculated using the binomial confidence intervals for daily proportion of visitors and are scaled with the population of each region of residence.

**Figure.**
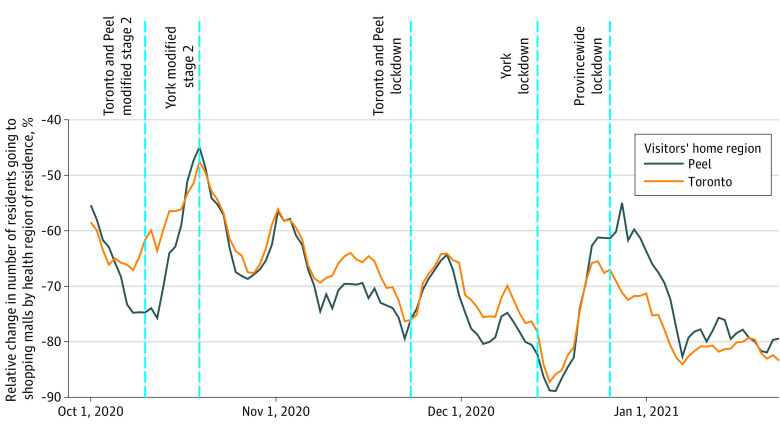
Visits From Toronto and Peel Residents to Shopping Malls in York, Relative to a Baseline Calculated for Each Day of the Week From January 1 to February 5, 2020 Toronto and Peel entered lockdown on November 23, 2020. York entered lockdown on December 14, 2020. Other regions entered lockdown on December 26, 2020.

## Discussion

Lockdowns in the urban core were associated with reduced overall visits to shopping malls and restaurants by residents but were not associated with decreased travel to these businesses in peripheral regions, where restrictions permitted indoor dining and shopping for nonessential businesses. We observed a large increase in visits to shopping malls in the peripheral regions by residents of the urban center in the week following the lockdown. These heterogeneous restrictions may lead to unintended consequences, undermining lockdowns in the urban core and driving residents from zones of higher transmission to zones of lower transmission. While our sample was limited to a fraction of the population, neighborhood sociodemographic characteristics were similar to the general population. Regional nonpharmaceutical intervention frameworks could avoid these consequences by implementing restrictions spanning both the core and periphery of urban areas or using interregional travel restrictions. These concerns are likely generalizable to other major metropolitan areas, which often comprise interconnected but administratively independent regions.^6^

## Notes

Supplement. **eFigure. **Map of the 5 Regions Comprising the Greater Toronto Area **eTable.** Neighborhood Sociodemographic Characteristics by Dissemination Area of Toronto and Peel Residents Included in the Veraset Mobile Device Sample
