# Supplementary material for: Trends in Interregional Travel to Shopping Malls and Restaurants Before and After Differential COVID-19 Restrictions in the Greater Toronto Area
Source: JAMA Netw Open. 2021 Aug 31;4(8):e2123139. doi: 10.1001/jamanetworkopen.2021.23139 (PMC8408668; doi:10.1001/jamanetworkopen.2021.23139)
Supplement: Supplement. — eFigure. Map of the 5 Regions Comprising the Greater Toronto Area eTable. Neighborhood Sociodemographic Characteristics by Dissemination Area of Toronto and Peel Residents Included in the Veraset Mobile Device Sample [file jamanetwopen-e2123139-s001.pdf]

Supplemental Online Content

Soucy JPR, Ghasemi A, Sturrock SL, et al. Trends in interregional travel to shopping malls and restaurants before and after differential COVID-19 restrictions in the Greater Toronto Area. *JAMA Netw Open*. 2021;4(8):e2123139. doi:10.1001/jamanetworkopen.2021.23139

eFigure 1. Map of the 5 Regions Comprising the Greater Toronto Area

eTable 1. Neighborhood Sociodemographic Characteristics by Dissemination Area of Toronto and Peel Residents Included in the Veraset Mobile Device Sample

This supplemental material has been provided by the authors to give readers additional information about their work.

© 2021 Soucy JPR et al. *JAMA Network Open*.

eFigure. Map of the 5 Regions Comprising the Greater Toronto Area

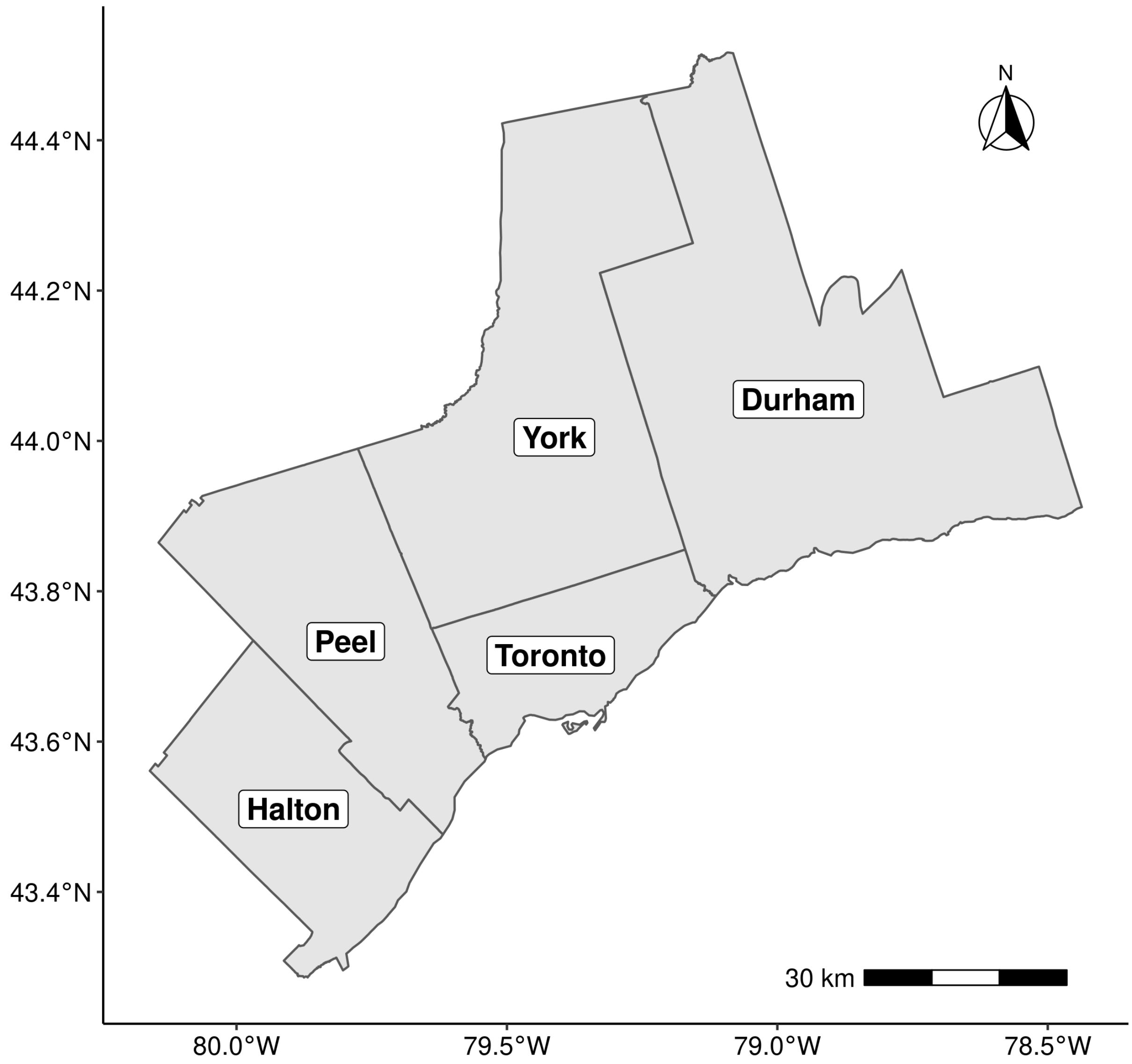

© 2021 Soucy JPR et al. *JAMA Network Open*.

eTable. Neighborhood Sociodemographic Characteristics by Dissemination Area of Toronto and Peel Residents Included in the Veraset Mobile Device Sample[a]

	Toronto			Peel		
	**Visitors**	**Full sample**	**General population**	**Visitors**	**Full sample**	**General population**
**Labor force characteristics**						
Percentage of essential workers	44.8 (31.8, 58.4)	46.5 (32.3, 59.0)	45.5 (31.5, 58.8)	50.7 (43.5, 58.5)	51.2 (43.6, 58.5)	52.5 (44.1, 60.4)
**Immigration and race**						
Percentage visible minority	48.5 (28.8, 73.6)	49.9 (27.4, 74.0)	50.7 (27.3, 75.5)	70.7 (42.2, 83.7)	70.1 (41.1, 83.0)	68.9 (42.7, 83.2)
Percentage recent immigrant	5.6 (2.6, 9.4)	4.8 (2.2, 8.8)	5.3 (2.4, 9.7)	5.7 (3.0, 9.6)	5.8 (2.9, 9.1)	6.0 (2.9, 9.4)
**Socioeconomic status**						
Percentage low income	16.3 (11.0, 26.3)	15.7 (10.7, 25.2)	16.8 (10.7, 27.5)	10.5 (7.4, 14.5)	10.2 (7.2, 13.8)	10.1 (7.5, 16.0)
**Housing**						
Average household size	2.6 (2.1, 3.0)	2.7 (2.2, 3.1)	2.6 (2.1, 3.0)	3.4 (2.9, 3.8)	3.4 (2.9, 3.8)	3.3 (2.9, 3.8)
Percentage multigenerational	19.5 (9.0, 32.3)	18.3 (8.6, 31.8)	18.3 (8.5, 32.4)	11.0 (5.9, 26.0)	12.0 (6.1, 26.9)	11.9 (6.2, 25.6)
Unsuitably crowded housing	8.9 (5.3, 16.4)	8.8 (4.7, 16.0)	9.5 (5.3, 17.7)	8.5 (5.4, 12.8)	8.2 (5.3, 12.5)	8.6 (5.3, 13.8)

[a] Neighborhood sociodemographic characteristics by dissemination area of Toronto and Peel residents included in the Veraset mobile device sample (mall and restaurant visitors and the full sample, visitors, and nonvisitors) vs the general population (all dissemination areas) from November 16, 2020, to November 29, 2020. Median values shown (interquartile range, IQR, in brackets). Total samples sizes are 154,089 (full sample) and 11,527 (mall and restaurant visitors). Each dissemination area represents approximately 400 to 700 residents. All sociodemographic data were obtained from the 2016 Canadian census. Veraset collects its mobility data from a variety of third-party providers such as mobile application developers. The median reported location accuracy in this dataset was 11.8 m.

© 2021 Soucy JPR et al. *JAMA Network Open*.
